# Prevention of Adolescent Depression in the Spanish-Speaking World

**DOI:** 10.3390/ijerph110605665

**Published:** 2014-05-27

**Authors:** Andrea B. Horn, Catalina Cañizares, Yvonne Gómez

**Affiliations:** 1 Department of Psychopathology and Clinical Intervention, University of Zürich, Binzmuehlestr. 14/17, 8150 Zürich, Switzerland; E-Mail: a.horn@psychologie.uzh.ch; 2 Department of Psychology, University of Los Andes, Carrera 1 Este Nr. 18 A-12, Bogotá, 11001, Colombia; E-Mail: c.canizares54@uniandes.edu.co

**Keywords:** depression, adolescence, prevention, Spanish-speaking depression programs, review

## Abstract

This paper aims at presenting programs targeted at the prevention of adolescent depression applied with Spanish-speaking populations that have been developed in Spanish-speaking countries and are mostly published in Spanish. These programs have been developed under different cultural contexts in Spain and Latin-America. The main goal of this paper is to make the studies and movements of the Spanish-speaking literature in this field accessible to the non-Spanish-speaking part of the research community. Therefore, after an introduction referring to possible cultural differences regarding depression in general and epidemiological basics, several programs are introduced. In total 11 programs will be shortly presented and discussed. After revising the programs it can be concluded that in the Spanish-speaking world many programs have been developed and conducted following current state of the art-approaches for adolescent depression prevention. Further research is needed especially targeting possible cultural and contextual aspects of prevention measures and their efficacy and efficiency.

## 1. Introduction

Depression is a major public health problem worldwide, as it shows high prevalence and leads to disability and individual suffering [1]. As depression often starts in adolescence, this developmental period is a critical one, because the likelihood of onset increases with puberty and the risk of recurrence is higher in younger individuals [2]. As a result, there is a broad consensus that prevention optimally should take place before the disease occurs in the first place.

All this is also true for the hispanophone sphere: more than 500 million individuals in the world speak Spanish, therefore it is the second language in the world by number of speakers and the second language for international communication [3]. Twenty five countries around the world have Spanish as an official language, most of them on the American continent. There are estimations that by 2050 the United States of America will be the country with the biggest Spanish-speaking population in the world [3].

This article aims at presenting prevention programs targeting adolescent depression that are applied to Spanish-speaking populations that have been developed in Spanish-speaking countries and are mostly published in Spanish. There is a conceptual focus on cognitive-behavioral prevention techniques. Making the programs available to a broader public seems worthwhile as it fosters cross-cultural exchange in the area of public health, and might inspire new clinical and research strategies. However, this article might not represent all research projects in this field conducted in the hispanophone world. There are several reasons for this: one main reason is the fact that there is no Spanish research database that includes all journal articles as well as master and doctoral theses, and other kind of scientific publications. Furthermore, the reality within the Spanish-speaking research community is not equivalent to the English-speaking publication culture. Many research projects are not published in international journals but in books and local journals not covered by the common databases. The attempt was made to broaden our search strategy by contacting experts; however, pretending to have covered the wide range of research activities in the field of the many Spanish-speaking countries might be presumptuous. Nevertheless, the research presented in this article might still serve the purpose to at least present a significant part of the Spanish-speaking literature.

The article is structured as follows: first, some short remarks regarding prevalence of depression and discussion regarding cross-cultural aspects of depression will be given. After introducing our search strategy, we will first present programs applied to Spanish-speaking populations that have been evaluated. Then, we will present programs that are currently work in progress, and finish with a discussion of the presented results.

### 1.1. Depression in Spanish-Speaking Populations

The prevalence of depression among Spanish-speaking adolescents is comparable to what is reported in the rest of world. Namely, prevalence rates reported in adolescent populations range from 10.3% in Spain [4] and 17.09% in Colombia [5] 14.3% in Chile [6] to 26% in Argentina [7]. These studies report as risk factors for developing a major depression episode in adolescence female sex, violence, conflict in the family, and low socioeconomic status.

The treatment of child and adolescent depression has also been covered widely in the hispanophone literature. A meta-analysis on the effects of psychotherapy on child and adolescent depression published in Spanish including fifteen controlled trials carried out between 1980 and 2002 revealed effect sizes of d = 0.53 for after the treatment and an effect size of d = 50 follow-up [7]. However, two thirds of the included studies were conducted in the United States and the other third consisted of studies conducted in Canada, United Kingdom, Puerto Rico and Australia. The only hispanophone research included was a Puerto Rican study by Roselló and Bernal [8]. The program used in this study is based on “The Depression Prevention Course” as introduced by Muñoz *et al.* [9] which was developed in the course of the San Francisco Depression Prevention Research Project with a strong focus on the inclusion of minorities in treatment research. For that purpose the Latino Mental Health Research Program was implemented [9] aiming at developing bilingual (Spanish/English) mental health manuals and testing them empirically. The “Depression Prevention Course” is based on social learning theory and relies on cognitive and behavioral methods. Research by Bernal and his colleagues in Puerto Rico [10] supports the feasibility of a culturally adapted version of the San Francisco General Hospital (SFGH) Depression Clinic manual in Latin American settings and showed effects in an adolescent sample [9]. Being interested in the external and ecological validity and culturally sensitive research, the authors developed a cultural sensitivity framework that could be of use to clinical researchers either in developing new or adapting existing treatment manuals to Hispanic populations [10]. The proposed model aims at increasing ecological validity in treatment manuals when working with different cultures. The framework differentiates eight dimensions: language (*i.e.*, vocabulary is clear and understandable), person (therapeutical relationship: role of ethnic/racial similarities, and differences between client and therapist), metaphors (symbols and concepts), content (the use of case example that reflect values and traditions from the treated group), concepts (treatment concepts concordant with culture and context), goals (treatment goals consonant with cultural expectations), methods (cultural adaptation of treatment methods), and context (considerations for contextual issues) [10]. Further elaboration of this topic would exceed the purposes of this article; more conceptual reflections on cross-cultural—adaptation can be found consulting Bernal, Jimenez-Chafey, and Domenech Rodríguez [11]. The model was applied in adapting US-American treatment manuals [10] to a Puerto Rican adolescent sample in two randomized controlled design studies [8,9,10,11,12]. Adolescents meeting the criteria for major depression were referred from schools in San Juan, Puerto Rico, and benefited significantly from participating in the program.

In summary, even if there are considerable cultural and societal differences regarding the conceptualization of depressive mood, the phenomenon of depression in general and adolescents’ depression in particular seem to be comparable across cultures. Adolescent depression is a common problem as relevant in the hispanophone populations as in the rest of the world. Therefore, evaluating and implementing prevention programs suitable for different Spanish-speaking populations is an important task from a general public health perspective.

### 1.2. Spanish Review Publications on Prevention of Depressive Symptoms in Adolescence

The interest in prevention of adolescent depression is also significant in the Spanish-speaking literature. This is reflected in several literature reviews and reflections on that topic that have been published. For example, a study with Argentine adolescents focused on anxiety and depression symptoms; the aim was to develop a profile of prevention measures needed on the basis of adolescents’ needs as assessed in surveys and group sessions similar to focus groups [13]. Furthermore, Cova, Aburto, Sepulveda and Silva published [14] a review on depression prevention programs*.* They highlight that a remarkable number of programs have been developed and evaluated to prevent children and teenager depression with promising results. However, all the revised programs took place in developed countries in English. The authors underline the importance of increasing research in this area in the reality of Spanish-speaking developing countries in Latin-America. Accordingly, they discuss the difference between efficiency of very expensive controlled trials and the lack of effectiveness in terms of possible implementations in the Latin-American reality. They supported the inclusion of psychosocial factors like poverty, ethnicities, rural *vs.* urban populations, cultural differences, e.g., regarding gender roles. Furthermore, they suggest fostering less costly dissemination of prevention programs, e.g., by training of non-psychologists like teachers as facilitators of prevention programs. Moreover, Martinez, Rojas and Fritsch [15] conducted a literature review about school-based depression prevention programs for adolescents and reported 6 established programs developed in the English language and one program developed by the authors themselves in Chile that they are currently evaluating. This program will be presented later in this article.

## 2. Method

The search for published studies on prevention programs for adolescent depression was performed in steps: first, the data bases Psicodoc, Dialnet Redalyc and LILACS, including the most important Spanish journals were researched with the key words “adolescents”, “prevention”, “depression”, and “cognitive-behavioral therapy” (in Spanish: adolescentes, depresión, prevención, programas de intervención, terapia cognitivo-conductual). Original articles were considered if they included prevention programs with depression prevention as a primary goal; programs with further goals than depression prevention were also included. However, only reviews in Spanish mostly covering English literature resulted from this search but no original study. As a second step, Spanish language meta-analyses, studies on risk factors, and review papers on prevention of depression in the adolescent population were reviewed. Thirdly, the index of important journals in the field as reflected in the review articles (Salud Mental, Psicothema, Psicología Conductual, Revista Latinoamericana de Psicologia) were analyzed separately. Furthermore, the experts appearing in the literature were additionally contacted by e-mail and asked to send information about their own research and colleagues researching in the field. By doing so, we also identified a number of programs that are being researched in ongoing research projects, but only preliminary results are available in congress publications as sent to us by the authors.

In the end, these research strategies yielded five Spanish-speaking prevention programs, which have been studied in randomized controlled trials and conducted, and published in Spanish, and a sixth one, that has been published in English. Furthermore, five ongoing trials were detected with preliminary results that also seemed worthwhile reporting.

## 3. Results

The following sections aim at presenting prevention programs that have been conducted in Spanish by Spanish-speaking research teams in Spanish-speaking countries. All studies presented in this section have a non-treated, randomized control group and published result data. In contrast, in the last part of the section, a couple of ongoing studies will be shortly presented that do or do not have a control group or results have not yet been reported. All presented studies are summarized in Table 1.

**Table 1:** Included prevention studies.

Program	Study	Target population	Country where implemented	Sessions/ outcome	*N*	Follow up	Results
Improvement of self-concept and affective state	Mestre, V & Frias, MD (1996) [16]	Adolescents. Average age 12.5 years.	Spain	17, twice weekly; 30 to 60 min/CDI; EAC; STAIC; S-1.	*N* total = 241; prevention group *N* = 125 control group *N* = 110	NO	Positive effects on proximal variables—self-esteem-particularly in adolescents aged 12 years and older.
Coping with emotional problems in adolescents	Olmedo, M., Del Barrio, V. & Santed, MA (2003) [17]	Adolescents between12 to 16 years old	Spain	18 weekly/ CDI-S; STAIC; Academic performance.	*N* = 121	Pretest and posttest	Differential effectiveness of the program for adolescents with academics problems. The group with academic problems showed more pronounced changes in depression scores
LISA PD	Gomez, Y., Restrepo, V. & Jiménez, JC (2004) [18]	Adolescents aged between 12 and 16 years old (7th grade)	Colombia	9 weekly/ CES-D; ATQ-RP; PANAS; WBSI.	*N* = 147	Pretest and posttest	The differences between control and treatment group were only marginally significant.
EDUPEC	Gómez, Y., López, P. & Jiménez, G. (2010) [19]	Adolescents aged between 14 and 17 years old (8th Grade)	Colombia	6 weekly/ ATQ-RP.	Experimental group *N* = 107; control group *N* = 83	Pretest and posttest	They found statistically significant differences between groups only for negative thoughts.
Emotional wellbeing workshop for teens	Cova, F., Rincón, P. & Melipillán, R. (2011) [20]	Female adolescents	Chile	11 (90 min)/ BDI-II; DISC-IV; BAI; BAI; ACSQ; RRS; Rosenberg Self-Esteem Scale.	Experimental group *N* = 119; control group *N* = 118	Pretest and posttest	The program did not show effects in any of the two modalities. The indicated modality obtained more satisfaction with participation.
FRIENDS for Life	Gallegos, J., Linan- Thompson, S., Stark, K., & Ruvalcaba, N. (2013) [21]	Adolescents aged between 8 and 13 years old.	Mexico	10 weekly/ SCAS; CA; CDI.	*N* = 103	Pretest and posttest and 6 months later.	The program showed a positive effect it reduced symptoms and risk factors of depression. As well, it increased proactive coping skills.
I think, I feel, I act	Araya, R., Fritsch, R., Spears, M., Rojas, G., Martinez, V., Barroilhet, S., Vöhringer, P., Gunnell, D., Stallard, P., Guajardo, V., Gaete, J., Noble, S. & Montgomery, A. (2013) [22]	Adolescents with an average age of 14.5	Chile	12 sessions BDI-II; RCADS; CATS;	*N* = 2500	Pretest and posttest Follow up: 3 and 12 months.	No evidence of clinically differences in depression scores between the groups.
Psycho-education for parents aimed at preventing affective problems.	Barcelata, BE., & Gómez, E., (2006) [23]	Parents	Mexico	6 (120 min)/ ECPSE.	*N* = 67	Pretest and Posttest	The preliminary study yielded results showing statistically significant differences between pre and post treatment
EMAS: Strategies to maintain positive mood.	Saez, E., Bonilla, K., Galloza, A. & López, C, (2009) [24]	Adolescents	Puerto Rico	Unspecified	Unspecified	Unspecified	Final results have not been published yet.
Smile	Sánchez, O, Méndez , FX. & Garber., J., (2009) [25]	Adolescents	Spain	4 modules/ ACSQCMAS-R; RCQ.	Unspecified	Unspecified	Final results have not been published yet
Fortius	Mendez, FX., Espada, JP & Amoros, MO., (2012) [26]	Adolescents	Spain	12 weekly/	Unspecified	Unspecified	Results have not been published yet
How to be adolescent today and not die trying?	Cingolani, J. (2009) [27]	Adolescents from 15 to 18 years old	Argentina	12 (90 min)/ Control sintomático; resiliencia y bienestar psicológico; satisfacción con la vida.	*N* = 70	3 and 6 months	Final results have not been published yet

Notes: Outcome measures as mentioned in the publications: CDI: Children Depression Inventory; EAC: Cuestionario de Autoevaluación autoconcepto; STAIC: State Trait Anxiety Children; S-1: Evaluación de eventos estresantes; CDI-S: Children Depression Inventory Short Form; CES-D: Center of Epidemiological Studies Depression; ATQ-RP: Automatic Thoughts Questionnaire Revised Positive; PANAS: Positive and Negative Affectivity Scale; WBSI: White Bear Suppression Inventory; BDI-II: Beck Depression Inventory; DISC-IV: The Diagnostic Interview Schedule for Children; BAI: Beck Anxiety Inventory; ACSQ: Adolescent Cognitive Style Questionnaire; RRS: Ruminative Response Scale; SCAS: Spanish version of the Spence Children’s Anxiety Scale; CA: Cuestionario de Afrontamiento; RCADS: Revised Child Anxiety and Depression Scale; CATS: Children’s Automatic Thoughts Scale; SPSI-R: Social Problem-Solving Inventory-Revised; ECPSE: Escala de Creencias para Padres sobre Salud Emocional; CMAS-R: Children’s Manifest Anxiety Scale Revised; RCQ: The Readiness to Change Questionnaire.

### 3.1. Programs Conducted in Spanish with Published Results

#### 3.1.1. Programa de Intervención Para Mejora del Autoconcepto y Estado Emocional (Improvement of Self-Concept and Affective State)

This program aims at improving self-esteem and preventing depressed and anxious states in adolescents between 11 and 14 years of age and was developed and implemented in Valencia, Spain [16]. The program is based on cognitive-behavioral techniques, targeted at a general school-population and has the following contents: first, evaluation of problem situations in the school setting is performed. The second part is dedicated to anxiety reduction facing stressful situations. This goal is pursued by training techniques regarding relaxation, impulse control, self-instructions, and improved communication. The third part aims at improving problem solving skills. The next sections it dedicated to affective learning strategies. Furthermore, constructive reappraisal of stressful situations is trained. In the last part, a review on the program is given and reflected where expectations were met and progress is observable and where expectations and goals need to be adapted to the realistic progress made. In total, the program consists of 17 sessions.

In 1996, a study was published by Mestre and Frias [16] implementing the program group by group in a school-setting including 12 groups within eight different schools in Valencia, Spain. The adolescents, aged on the average 12.5 years, were randomized group by group into the prevention or the non-treated control group. In total, 241 students took part in the study, 125 in the prevention, and 110 in the control group. The program was facilitated by five licensed psychotherapists in the course of three to four months, with two weekly sessions of 30 to 60 min length. Before and after the program, baseline and post-measures of self-esteem, helplessness, performance anxiety, anxiety, and depression were assessed.

The depression score of the CDI did show an improvement after the program; however, the control group did improve as well, so that there was no significant observable effect of the program. Nevertheless, self-esteem improved significantly in the treated group in comparison with the non-treated control group. Moreover, a significant interaction between age and treatment condition was observed: students older than 12 years (about half of the sample) benefited more from the program than the younger ones. No sex differences in terms of program effects could be observed. Unfortunately, none of the analyses took into account the nested data structure of each individual belonging to a school group. The authors conclude that the program had the planned positive effects on proximal variables—here self-esteem—particularly in adolescents aged 12 years and older. They argue that adolescents in this age are more prone to affective symptoms and thus suggest the age of 12 and above as ideal for the application of prevention programs.

#### 3.1.2. Programa de Afrontamiento de Problemas Emocionales en Adolescentes (Coping with Emotional Problems in Adolescents)

Olmedo, Del Barrio, and Santed [28] developed in Córdoba (Spain) a school-based program that consists of 18 weekly sessions in a group context. The program is based conceptually on a coping framework and aims at improving coping skills relying on cognitive-behavioral techniques in a general adolescent population. The program aims at fostering functional and replacing dysfunctional coping strategies. It consists of four modules: (1) improvement of self-esteem; (2) relaxation strategies; (3) improving social skills; and (4) improvement of problem solving skills. The first module consists of five sessions including self-observations tasks and cognitive restructuring. Relaxation techniques autogenic training and progressive muscle relaxation as well as imaginative strategies are introduced in the second module, which consisted of four sessions. The third section is mainly targeted at the training of assertive aspects of social skills during five sessions. Finally, planning and problem solving is the topic of the remaining four sessions, introducing techniques as suggested by Spivack and Shure [18] like mean-ends, alternative solution, and consequential thinking, or weighing pros and cons. Unfortunately, in both published studies the authors do not mention who actually implemented the training—e.g., teachers or psychologists—so that it cannot be concluded which training the facilitators of the course had.

The authors underline the advantage of implementing the program at schools, as the school setting represents a reference point where adolescents form their self-images being exposed to performance related, and competitive situations as well as affiliative processes. They also pointed out the fact that the school setting is the place where academic as well as social status is determined and thus typical risk factors for depression begin [28].

Two studies have been published so far, empirically testing the program: In the first study [28] 225 adolescents with an average age of 13.2 participated (the N of treatment *vs.* control group is unfortunately not mentioned). The intervention took place in public schools in Córdoba, Spain. The participants were group by group randomized to the prevention or to a non-treated control group. All students answered questionnaires before, as well as one week and five months after the intervention.

The two groups did not differ at any baseline rating. At baseline, the authors conducted several hierarchical regression analyses, showing the expected associations between the protective factors self-esteem, social skills, and interpersonal problem solving, and depressive symptoms as well as the anxiety measure. They interpreted this as empirical support for the chosen content of the prevention program. T-tests conducted comparing the post scores between treatment and control group show significant differences as expected, while the protective factors showed increased scores, compared to the control group. Furthermore, hierarchical regressions performed with change scores (pre-post) confirm, that the change in self-esteem and problem solving skills predicts the change of the amount of depressive symptoms after the prevention program.

Regarding the methods presented in the article, there are some caveats: first, as the treatment was conducted group by group the statistical analysis should take the nested data structure into consideration, which was not the case in this study. Secondly, the treatment effects in the comparison between treatment and control group were assessed without controlling for base line scores. Thirdly, no effect sizes are reported. However, the big sample, consistent results and the analysis including protective factors as mediating the effect on depression underline the validity of the study results.

A second study [17] was executed focusing on the role of academic performance as predictor of the effects of the program. Accordingly, no control group was involved but the differential effects of academic performance on the results of the prevention program explored. The sample consisted of 121 students of public schools in Córdoba aged between 12 and 16 years. The sample was divided into two groups: one without academic problems, and a second one with academic problems as manifested in the failing of at least one school subject. Unfortunately, the group size of the two groups is not reported in the article. The results indicated a differential effectiveness of the program for adolescents with academic problems: the group with academic problems showed more pronounced changes in their depression scores than the group without academic problems. The authors conclude that the association between academic performance and depression in adolescents is a highly relevant one, which should be considered in the area of prevention research. Furthermore, they underline that the effects can be bidirectional: depressive symptoms may lead to academic problems as well as academic problems can result in stressors preceding a heightened level of symptoms.

#### 3.1.3. LISA-T and EDUPEC

A further group-based cognitive-behavioral prevention program has been developed by Gómez and colleagues in Colombia [29]. It is based on the German program LISA-T, which is a universal school- based program aimed at preventing depressive symptoms in adolescents, and an earlier version of the program LARS&LISA developed by Pössel, Horn, Seemann and Hautzinger [30]. Gómez [29] translated the program and adapted it for a Colombian adolescent population. The cultural adaptation consisted, e.g., in changing the cover stories by substituting different examples in the program by ones taken from the everyday life reality of Colombian adolescents. Furthermore, some terms were not translated literally but were slightly changed in order to fit the program to the target sample. The content structure of LISA-T was maintained. The modules were: Introduction, Think (Cognitive restructuring), Do it (Assertiveness training) and Dare (social sills establishing new social contacts).

A study was conducted with a sample of *N* = 147 adolescents in Medellín, Colombia aged between 12 and 16 years old, who were in seventh grade. The classrooms were randomly assigned to the experimental and the control groups. Short (directly after finishing the program) and middle term (six months after) changes in following variables were measured: depressive symptoms positive and negative automatic thoughts, positive and negative emotions and suppression of thoughts base-line. Results indicated higher symptom levels in female than in male adolescents, as reflected in heightened level of depressive symptoms, negative automatic thoughts, negative affect and thought suppression. Students’ depressive symptoms were reduced from the baseline to the post assessment of depressive symptoms. The differences between control and treatment group were only marginally significant demonstrating reduced depressive symptoms for the experimental group [18]. In sum, the authors conclude that the program can be applied in the Colombian setting. The adolescents gave a positive qualitative feedback about the program, but uncontrolled variables such as the discipline problems of the seventh grade were present and might play a role in the results.

In a further study, the program was reformulated and only the cognitive part was applied as a singly module named Educación en la cadena pensamiento-emoción-conducta (EDUPEC—Education in the chain think, emotion and behavior) [19]. Again, the examples in the program were culturally adapted to the everyday life of Colombian adolescents in public schools of an urban area and referred to family or school related situations with affective and personal significance. The focus of the program is to identify dysfunctional information processing in these everyday life situations, particularly automatic thoughts. Consequently, once dysfunctional cognitive patterns are individually detected, basic cognitive techniques as distancing oneself from negative thoughts, and substituting them with more functional alternative thoughts are introduced and trained. Conceptually, this is presented to the students in a coping framework with the aim of enhancing functional cognitive coping strategies. The stress and coping-framework is especially adequate for universal programs as it is suitable to students without any symptoms as well as for adolescents already at risk. The six sessions are titled as follows: (1) starting the process to detect thoughts—feelings-behavior; (2) How are thoughts-feelings, and behavior related? (3) Identifying my dysfunctional and negative thoughts; (4) Questioning my dysfunctional and negative thoughts; (5) My new thoughts (functional and positive); and (6) Choosing my copings style.

This program was evaluated with 190 students aged between 14 and 17 years in public schools, whose classrooms were randomly assigned to an experimental or a control group. 53.31% (*n* = 107) formed the experimental group and 43.68% (*n* = 83) the control group. In the experimental group 44.85% (*n* = 48) were female and 40.93% (*n* = 34) in the control group. The program was applied in six weekly sessions of 45 min each run by trained psychologists; school teachers were not present. As the program was mainly targeted at cognitive processes the main outcome variable was the Automatic Thoughts Questionnaire.

Results indicated that actually negative automatic thoughts show a significant decrease after the program as compared to the control group and controlled for baseline (F = 5736, *p* = 0.018) [19]. No changes regarding positive automatic thoughts could be observed. Effect sizes are not reported.

#### 3.1.4. Taller de Bienestar Emocional Para Adolescentes (Emotional Wellbeing Workshop for Teens)

Cova, Rincón and Melipillán [20] developed a prevention program for female Chilean adolescents based on Clark and Lewinsohn’s Adolescent Coping with Stress Course, Gilliham’s APEX Program, and the Resourceful Adolescent Program by Shochet’s group. The complete program has 11 sessions that last 1 h and 30 min each. The modules include the comprehension of the relationship of emotions, cognitions and behaviors as well as the enhancement of communication abilities, coping, emotional awareness, and cognition control.

The program was implemented in two modalities: the indicated form was applied to teenage girls with sub-threshold depressive symptoms (experimental group = 101; control group *N* = 108) and the universal application to complete classes (experimental group = 119; control group *N* = 118). The program did not show significant effects on depressive symptoms, anxiety, self-harming or externalizing behaviors in any of the two modalities as compared to a non-treated control group. While focusing only on the constructive cognitive reflection was there a significant positive effect compared to the control group. Moreover, as in other studies in the indicated modality participants reported more satisfaction than in the universal condition. However, one quarter of the female teenagers participating in the targeted program reported being irritated by the fact that they participated in the program, a reaction that was reported in no more than 6% of the universal conditions. In sum, the authors conclude that dose and scope of prevention programs were not adequate for the target population.

#### 3.1.5. FRIENDS for Life

“FRIENDS for Life” [21] is a program developed in Australia which has been adapted to cultural aspects of Latin American children and translated into Spanish by a research group in México. The program is designed to enhance coping strategies and emotional skills for stressful situations and thus to prevent depression and anxiety. Accordingly, its scope is broader than just preventing depression. However, as depression prevention is one central goal of the program has been included in this compilation. Specifically, in the behavioral component of the program children are taught to monitor feelings and thoughts. Moreover, the cognitive factor includes recognizing feelings and the link they have with thoughts, as well as faulty cognitions and incompatible self statements. The ten weekly sessions included in the program contain learning techniques, like group discussions and role playing, in addition to following the seven steps represented in the acronym with the word FRIENDS [21].

Clinical trials with the English original program conducted by Barrett’s research team [21], have shown reductions in depressive symptoms. In México, the version of the program in Spanish language was delivered by teachers to 1030 children aged between 8 and 13 years old. Results show that the preventing program reduces risk factors and depressive symptoms just as it enhances proactive coping [21]. These findings suggest that a universal prevention program is an effective strategy to promote mental health. The fact that school teachers can deliver the intervention is an additional benefit that adds to the profitability of the strategy, because a large number of adolescents can benefit from the program.

Moreover, this is one of the first studies to examine prevention programs in adolescents in Mexico, as well as the first step to evaluate how preventive interventions should be delivered to developing countries taking into account the social and economic limitations. Hence, studies should evaluate the long-term effects of “FRIENDS for Life” as a tool to promote emotional resilience within the class-rooms, since in the current study only short-term effects were reported [21].

#### 3.1.6. Yo Pienso, Yo Siento, Yo Actúo (I Think, I Feel, I Act)

A big research project from the University of Chile and Bristol University under the direction of Araya and financed by the Wellcome Trust is being implemented as a school-based intervention to improve the mental health of low-income, secondary school students in Santiago, Chile [22]. The research group has created a program named “I think, I feel, I act”. The program consists of 11 sessions organized as follow: one session to give an introduction and explain the influence of thoughts about what I feel and how I act; six sessions dedicated to how I think, the pessimistic thinking style, how can I change and to learn emotional regulation; three sessions centered in problem solving and the last session to summarize what was learned before and how it can be applied it in the future [22].

Fritsch [31] reported first data with an adjusted version of the program after the pilot study based on 181 students from four classes: qualitative feedback by the participating students lead the authors to the conclusion that the program is feasible to implement, and well accepted and perceived as useful by students.

Furthermore, the program has been applied approximately to 2500 students with an average age of 14.5 years in 22 schools in Chile [22]. The trial showed that the intervention was not significantly better than usual care, although the program was based on evidence, had good levels of attendance, used robust methodological aspects, presented high levels of adherence, and a reasonably long follow-up period. Given these results, the authors point to the evidence of other studies showing that universal school-based interventions tend to display small to none effects on depressive symptoms [22].

### 3.2. Spanish Speaking Prevention Programs: Work in Progress

As reported in the Method sections, our search strategy included contacting known experts in the field by e-mail. Accordingly, beside the published studies we identified ongoing studies on adolescent depression prevention. As the programs are interesting and the studies promising we considered it worthwhile to also briefly mention those projects that are still in progress and have not been published yet. These studies are as also summarized in Table 1.

#### 3.2.1. Psycho-Education for Parents Aimed at Preventing Affective Problems

Barcelata Eguiarte and Gómez–Maqueo [23] present a program targeted at the prevention of affective symptoms in adolescents via psychoeducation of their parents by teaching them to identify signs of emotional problems. The program consists of six weekly sessions of two hours each. Parents are informed about affective problems in adolescents, specific developmental aspects of adolescence, how to detect depression in their children, the associations between violence in the family and adolescent depression, and finally, about the association between depression and addictive behavior in adolescence. The preliminary study yielded results showing statistically significant differences between pre and post treatment. The authors conclude that establishing psychoeducation as a primary prevention strategy can be successfully used for various purposes, both in the clinical and in the educational field.

#### 3.2.2. EMAS: Estrategias Para Mantener un Ánimo Saludable (Strategies for Maintaining a Positive Mood)

Saéz has developed a school-based prevention program for depression in Latino pre-adolescents: Strategies for Maintaining a Positive Mood (EMAS) [24] based on previous trials conducted by Roselló, Bernal and, Rivera-Medina examining the efficacy of a cognitive behavioral therapy (CBT) and interpersonal psychotherapy (IPT) program for depression. The program is organized in 12 sessions held once a week provided in both group and individual formats. The main objectives are targeted at preventing the risk factors for depression by promoting problem solving skills, interpersonal relationships, and positive mood. The conducted trial included 112 adolescents referred from Puerto Rican schools with ages in between 12 and 18. The results reveal that the CBT condition yielded significant decreases in depressive symptom and improved self-concept compared to IPT. As well, there were no significant differences between the individual and the group formats in reducing symptoms of depression [32].

The innovative aspect of the EMAS program is that is has been integrated into the regular school curriculum and school teachers are trained to implement it. Also the format of the program was developmentally and culturally adapted to Puerto Rican adolescents. The ongoing study consists in evaluating the efficacy of the program in three schools in Puerto Rico using a classroom by classroom randomized study with pre/post measures and four follow ups. However, results have not been available yet. Nonetheless, a pilot study indicated that the program can be effectively delivered by teachers and was well accepted by students [33].

#### 3.2.3. SONRISA (SMILE)

Smile is a program developed by Sánchez and Méndez in Spain [25] based on the program PEAC (Programa Emoción-Actividad-Cognición: Emotion-Activity-cognition Program) for children by Méndez [34].

The program is organized in four modules: (1) development of curiosity and motivation to engage in the program; (2) Emotional education; (3) Behavior therapy (positive activation; problem solving; relaxation (breathing); interpersonal skills) and (4) optimism.

The novelty of the program is the emphasis on motivation and curiosity in order to encourage participation in the program, and the inclusion of elements of positive psychology. A preliminary study focusing on the increase of motivation and interest of adolescents to participate in programs for the prevention of depression was conducted. The results are promising regarding the usefulness of the pre-intervention. Furthermore, they found a negative correlation between curiosity about change and anxiety, and a positive correlation with optimism [25].

#### 3.2.4. FORTIUS

The program was created by Méndez, Espada, Orgilés at the University of Murcia, Spain [26]. The main objectives of this program, beside depression prevention, are to enhance psychological strength, to prevent emotional distress and build better abilities to solve problems appropriately. It focuses on emotions, behaviors and cognitions each of which is directed to understand the utility, function and control of negative emotions, as well as training in social abilities and cognitive restructuring. Moreover, the program integrates positive psychology oriented to personal development and wellbeing broadening the classical scope of clinical psychology that traditionally focuses on minimizing discomfort, and to overcome problems. The program includes 12 sessions plus one follow up session. The complete protocol contains precise instructions as well as abundant teaching material directed to adolescents aged between 8 to 12 years [26]. So far no studies have been published.

#### 3.2.5. Cómo ser Adolescente Hoy y no Morir en el Intento? (How to be Adolescent Today and not Die Trying?)

Referring to findings regarding the psychological vulnerability and healthy behavior of Argentinean adolescents [35,36] Cingoliani developed a psychosocial intervention program in 12 sessions to promote healthy development and prevent mood disorders [36]. The program is based on four general principles that are characteristics for the psycho-development dynamic for adolescents: stimulate coping strategies, promote emotional regulation, train interpersonal skills, and improve a responsible autonomy. The program is organized in 12 meetings of 90 min each implemented by two psychologists [27]. The program is at the point of being implemented and evaluated.

## 4. Discussion

This compilation of hispanophone literature on adolescent depression prevention shows that there are relevant contributions to field of prevention research that might hardly be acknowledged in the international research community. The studies refer to similar theoretical backgrounds and psychotherapy techniques as in the Anglo-Saxon literature. For example, the majority of the programs are based in cognitive-behavioral aspects of depression, showing that teaching certain behaviors and abilities lowers the risk factors and makes it less likely for an adolescent to develop depression [20] Furthermore, most programs are even based on established English speaking programs (e.g., Depression prevention course, FRIENDS). Many programs are multimodal and provided by psychologist with the exception of EMAS and FRIENDS, where the programs are disseminated by teachers in the school context. Referring to teachers as facilitators of prevention measures is a promising venue as it does not only save resources but also benefits from the familiarity the teachers have with the adolescent population and the school context. In earlier programs cognitive and behavioral techniques are most prevalent, more recent programs tend to include resource oriented fostering of wellbeing and emotional skills. Also motivational aspects have been targeted recently which seems promising as drop-out rates particularly in groups that are most in need of preventive interventions is an important issue. For the future, including new media based on internet or cell phone into prevention programs seems to be promising as it might require less resources and is highly accessible to adolescents even in marginalized populations [37,38].

Following the structure of cultural adaptation as introduced by Bernal and colleagues [11], there are several aspects that need to be considered when adapting therapy strategies to different cultural backgrounds: First, most obviously the language has to be changed. This is not as trivial as it sounds; if the translation is awkward even evaluated English or German speaking programs might not work, particularly not with adolescents. Also in terms of international research comparability, language can be a matter, as instruments need to be translated and evaluated in the language and cultural context. This might be a barrier for conducting studies, and for publishing it internationally. Personal background is the second dimension, which is suggested as being relevant. Example given, targeting a prevention program to a Mexican community living in the USA has different cultural implications than targeting it to adolescents in Mexico City: The migrant status and the cultural context differ and lead to different needs for cultural adaption. Additionally, *metaphors* and *content* needs to be changed often: e.g., social norms are culturally sensitive. What is seen as adequately assertive in the USA might be rude in Colombia. Therefore, behavioral intervention, e.g., targeting social skills need to be adapted, as it was done in most of the mentioned programs. Also *values* are culturally different, so life goals and thus even goals in prevention and therapy might be different. Interestingly enough, these adaptations are seldom mentioned in publications, which might be a bias: the more similarity to already evaluated programs is suggested, the easier it is published.

Many programs focus one the training of emotion regulation skills. This goes in line with recent “third wave” therapies that have been published. In terms of cultural differences, emotion regulation is a topic of particular interest. For example, the expression of emotions is a fundamental aspect of emotion regulation which is known to vary across cultures; it is assumed that there are different display rules of emotional expression depending on the cultural context [39], beside the suggested universality of basic emotional processes.

## 5. Conclusions

To sum up: many cultural aspects are expected to be highly relevant in depression prevention. However, beside private conversations this is hardly reflected in the sparse literature on non-English prevention trials. Following this further, the majority of the literature found in prevention focuses on the principal risk factors for depression and in the development of interventions, however clinical trials proving the efficacy of the programs in Latin American adolescents are often not published for an international audience. Another problem is that sometimes trials lack strong study designs, or programs focus on other life skills and the central variable is not depression, instead they include variables such as alcohol or psychoactive substances abuse [40,41]. Many of the Spanish-speaking countries have different economical and societal conditions as compared to the USA. Undoubtedly, research on the prevention of depression is mostly conducted in developed countries, since only 3% of the published research articles come from low and middle-income countries, especially when it focuses on children or adolescents [21]. This seems problematic as the social and financial constraints in many countries constitute risk factors for the development of mental health problems. As well, studies have shown that youth that are in greater risk for depression show larger effects in prevention programs [42].

To conclude, it is commonly agreed upon that there is a need for more evaluations of the running programs and a dissemination of the findings in international publications. However, the scientific literature on the topic and the presented programs reflect a deep integration in international mainstream and is in state of the art in terms of conceptual foundations, development, and dissemination of prevention programs for adolescents. In general, however, beside scientifically proven efficacy of the programs, efficiency, *i.e.*, the extent to which the programs achieve their intended effects in the usual clinical setting is far from being tested satisfactorily. Still, this review might show that important first steps are done in Spanish-speaking countries to overcome this gap.
